# Unanswered questions in the use of blood component therapy in trauma

**DOI:** 10.1186/1757-7241-19-5

**Published:** 2011-01-17

**Authors:** Steven R Allen, Jeffry L Kashuk

**Affiliations:** 1 Division of Trauma, Acute Care, and Critical Care Surgery, Department of Surgery, Penn State Hershey Medical Center, College of Medicine, Hershey, PA, USA

## Abstract

Recent advances in our approach to blood component therapy in traumatic hemorrhage have resulted in a reassessment of many of the tenants of management which were considered standards of therapy for many years. Indeed, despite the use of damage control techniques, the mortality from trauma induced coagulopathy has not changed significantly over the past 30 years. More specifically, a resurgence of interest in postinjury hemostasis has generated controversies in three primary areas: 1) The pathogenesis of trauma induced coagulopathy 2) The optimal ratio of blood components administered via a pre-emptive schedule for patients at risk for this condition, ("damage control resuscitation"), and 3) The appropriate use of monitoring mechanisms of coagulation function during the phase of active management of trauma induced coaguopathy, which we have previously termed "goal directed therapy". Accordingly, recent experience from both military and civilian centers have begun to address these controversies, with certain management trends emerging which appear to significantly impact the way we approach these patients.

## Introduction

As outlined by Dries [1], recent advances in our approach to blood component therapy in traumatic hemorrhage have resulted in a reassessment of many of the tenants of management which were considered standards of therapy for many years. Indeed, despite the use of damage control techniques, the mortality from trauma induced coagulopathy has not changed significantly over the past 30 years [2,3]. More specifically, a resurgence of interest in postinjury hemostasis has generated controversies in three primary areas: 1) The pathogenesis of trauma induced coagulopathy 2) The optimal ratio of blood components administered via a pre-emptive schedule for patients at risk for this condition, ("damage control resuscitation"), and 3) The appropriate use of monitoring mechanisms of coagulation function during the phase of active management of trauma induced coaguopathy, which we have previously termed "goal directed therapy". Accordingly, recent experience from both military [2] and civilian centers[3] have begun to address these controversies, with certain management trends emerging which appear to significantly impact the way we approach these patients.

### Pathogenesis of trauma induced coagulopathy

Coagulation disturbances following trauma appear to follow a trimodal pattern, with an immediate hypercoagulable state, followed quickly by a hypocoagulable state, and ending with a return to a hypercoagulable state. An improved understanding of the early hypocoagulable state, or "trauma induced coagulopathy", has received particular attention over recent years. This state was traditionally believed to be the consequence of clotting factor depletion (via both hemorrhage and consumption), dilution (secondary to massive resuscitation), and dysfunction (due to both acidosis and hypothermia). However, recent evidence documents the presence of a coagulopathy prior to fluid resuscitation and in the absence of the aforementioned parameters [4,5]. Specifically, coagulopathy was observed only in the presence of hypoperfusion (base deficit > 6) and was not related to clotting factor consumption as measured by prothrombin fragment concentrations. Furthermore, this state appears to directly correlate with thrombomodulin concentration [an auto-anticoagulant protein expressed by the endothelium in response to ischemia], and inversely correlated to protein C concentration. A decreased concentration of protein C also correlated with a decrease in the concentration of PAI, an increase in tissue plasminogen activator (tPA) concentration, and an increase in D-dimers. This final observation suggested that protein C-mediated hyperfibrinolysis via consumption of PAI may contribute to traumatic coagulopathy. The release of pro-inflammatory cytokines, in the presence of shock, likely results in two main perturbations of the coagulation system: (1) release of tissue factor with subsequent clotting factor consumption and massive thrombin generation, and (2) hyperfibrinolysis due to up-regulation of tPA. Specifically, diffuse endothelial injury leads to both massive thrombin generation and systemic hypoperfusion. These changes, in turn, result in the widespread release of tPA, leading to fibrinolysis. Both injury and ischemia are well known stimulants of tPA release, [6] and a strong correlation between hypoperfusion, fibrinolysis, hemorrhage, and mortality among injured patients who require transfusion has been noted [7].

Elucidation of the integral role of fibrinolysis also raises the possibility of mitigation of the coagulopathy via early administration of anti-fibrinolytic agents[8].

Although the endogenous coagulopathy of trauma results in an immediate hypocoagulable state among shocked patients following injury, several secondary conditions may develop, which exacerbate this pre-existing coagulopathy. Such conditions are, in large part, due to the complications of massive fluid resuscitation, and include clotting factor dilution, clotting factor consumption, hypothermia, and acidosis. Although these factors were considered traditionally as the driving force of traumatic coagulopathy, recent evidence suggests that their effect may have been overestimated [9,10].

Many causes of hypothermia exist for the trauma patient, including altered central thermoregulation, prolonged exposure to low ambient temperature, decreased heat production due to shock, and resuscitation with inadequately warmed fluids. The enzymatic reactions of the coagulation cascade are temperature-dependent and function optimally at 37°C; a temperature < 34°C is associated independently with coagulopathy following trauma [11]. Hypothermia also affects both platelet function [12] and fibrinolysis [13].

Clotting factor activity is also pH dependent, with 90% inhibition occurring at pH = 6.8 [14]. Coagulopathy secondary to acidosis is apparent clinically below a pH of 7.2. Because hypoperfusion results in anaerobic metabolism and acid production, it is difficult to discern the independent effect of acidosis on hemostatic integrity. Although the independent effect of acidosis on hemostatic integrity remains unclear, correction of acidosis via resuscitation remains a valuable therapeutic endpoint in terms of minimizing the aforementioned hypoperfusion-induced endogenous coagulopathy of trauma. Furthermore, maintenance of the arterial pH > 7.20 during resuscitation of shock (with bicarbonate, if necessary) maximizes the efficacy of both endogenous and exogenous vasoactive drugs.

In summary, an endogenous coagulopathy occurs following trauma among patients sustaining shock, and does not appear to be secondary to coagulation factor consumption or dysfunction. Rather, current evidence suggests that it is due to ischemia-induced both anticoagulation and hyperfibrinolysis. During this timeframe, therapy should focus on definitive hemorrhage control, timely restoration of tissue perfusion, and point of care monitoring.

### Damage control resuscitation

Consumption and dilution of clotting factors via crystalloid resuscitation and other factor-poor blood products perpetuates trauma induced coagulopathy. Coagulation factors present in plasma contained in PRBCs have minimal activity due to prolonged storage and associated short coagulation factor half-lives. Isolated administration of PRBCs in the absence of plasma will therefore potentiate the acute coagulopathy of trauma. Accordingly, most MT protocols advocate early replacement of factors and platelets. However, the definition of MT, and the timing and ratio of specific factor replacement, remains widely debated, due at least in part to differences in protocols as well as inherent flaws in retrospective data analysis. A valid definition of MT is lacking. The Denver group recently reviewed transfusion practices in severely injured patients at risk for post-injury coagulopathy, noting that >85% of transfusions were accomplished within 6 hours post-injury, suggesting this is the critical period to assess the impact of preemptive factor replacement, rather than the 24-hour time period frequently emphasized[9].

Current clinical MT protocols promoting "damage control resuscitation" (i.e., preemptive transfusion of plasma, platelets, and fibrinogen) assume that patients

presenting with life-threatening hemorrhage at risk for post-injury traumatic coagulopathy should receive component therapy in amounts approximating those found in whole blood during the first 24 hours. The U.S. military experience in Iraq [15] suggesting improved survival based on a 1:1:1 fresh-frozen plasma (FFP)-to-RBC-to platelet ratio has led to recommendations of fixed ratios of these blood products during the first 24 hours post-injury in civilian trauma centers[16].

Others, however, suggest that the optimal survival ratio appears to be in the range of a 1:2 to 1:3 FFP-to-RBC ratio [9]. It could be that the reported benefits from a 1:1 strategy likely represent a surrogate marker of survival. Specifically, those patients who survive injury are simply able to receive more plasma transfusions, as opposed to those who die from acute hemorrhagic shock early after injury.

The role of early platelet transfusion in the setting of hemorrhagic shock also remains debated. As with FFP, recent military reports have suggested routine administration of apheresis platelets to the injured patient. However, a similar survival bias has been suggested to explain the apparent benefit of early platelet administration.

Furthermore, studies from more than 2 decades ago evaluating clotting factor and platelet counts in massively transfused patients concluded that a platelet count of 100,000/mm3 is the threshold for diffuse bleeding, and that thrombocytopenia was not a clinically significant problem until transfusions exceeded 15 to 20 units of blood. Specifically, patients with a platelet count >50,000/mm3 had only a 4% chance of developing diffuse bleeding[17]. Although the classic threshold for platelet transfusion has been 50,000/mm3, a higher target level of 100,000/mm3 has been suggested for multiply injured patients and patients with massive hemorrhage. However, the relationship of platelet count to hemostasis and the contribution of platelets to formation of a stable clot in the injured patient remain largely unknown. Furthermore, platelet function, irrespective of number, is also of crucial importance. The complex relationship of thrombin generation to platelet activation requires dynamic evaluation of clot function. Accordingly, at this time, there is inadequate evidence to support an absolute trigger for platelet transfusions in trauma.

Concerns over high ratios of blood component therapy stem in large part from a growing body of evidence documenting the adverse effects of transfusion, as the association of massive transfusion of PRBCs with nosocomial pneumonia, acute lung injury, and acute respiratory distress syndrome (ARDS) has been well established[18]. These factors all suggest that monitoring of coagulation function with tailoring of treatment to the individual patient may improve our ability to administer blood component therapy in the acutely injured patient.

### Monitoring of coagulation function: Goal directed therapy

A major limiting factor of current MT protocols is the lack of a real-time assessment of coagulation function. Thromboelastography (TEG) may offer a real time visco-elastic analysis of the clotting process. First described by Hartert in 1948, [19] the technique utilizes whole blood in a rotating cuvette and heated to 37C. A piston is suspended in the sample and the rotational motion transferred to the piston as fibrin strands form between the wall of the curette and the piston. An electronic amplification produces a characteristic tracing to be recorded. TEG assesses clot strength from initial fibrin formation to clot retraction and finally in fibrinolysis. TEG has multiple advantages over other traditional assays of coagulation, as it provides information on the balance between the opposing components of coagulation, thrombosis and lysis. While the others are limited to a specific arm of the coagulation cascade and are less reliable in the hypothermic, acidotic trauma patient, TEG evaluates the entire clotting cascade as well as platelet function, and affords an improved clinical correlation of hemostasis to the cell based model [20].

**Figure 1:**
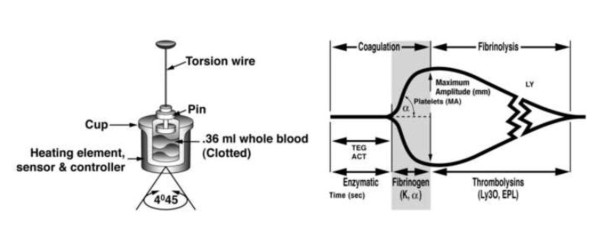
**Technique of Thrombelastography (reprinted with permission from Haemoscope Corporation, Niles, IL)**. (a) A torsion wire suspending a pin is immersed in a cuvette filled with blood. A clot forms while the cuvette is rotated 45°, causing the pin to rotate depending on the clot strength. A signal is than discharged to the transducer that reflects the continuity of the clotting process. The subsequent tracing (b) corresponds to the entire coagulation process from thrombin generation to fibrinolysis. The R value, which is recorded as TEG-ACT in the rapid TEG specimen, is a reflection of enzymatic clotting factor activation. The K value is the interval from the TEG-ACT to a fixed level of clot firmness, reflecting thrombin's cleavage of soluble fibrinogen. The α is the angle between the tangent line drawn from the horizontal base line to the beginning of the crosslinking process. The MA, or maximum amplitude, measures the end result of maximal platelet-fibrin interaction, and the LY 30 is the percent lysis which occurs at 30 minutes from the initiation of the process, which is also calculated as the EPL, or estimated percent lysis.

Goal-directed transfusion therapy guided by TEG tailors blood product administration to the physiological state of the patient. Using this technology, a variety of coagulation abnormalities have been noted that in the past would have been overlooked. With results available within 5 minutes, an initial hemostatic assessment with R-TEG identifies patients at risk for post-injury coagulopathy upon arrival. Blood component therapy is then tailored to address clotting derangements in a specific manner, and subsequent reassessment allows the evaluation of response until a set threshold is reached. This strategy also permits improved communication with the blood bank; based on initial assessment and response to component therapy, more accurate estimations of component requirements can be made [10]. Figure 1 depicts the various components of the TEG tracing, which enable a goal-directed approach to coagulopathy. Reflecting the initiation phase of enzymatic factor activity, a prolonged TEG-ACT value is the earliest indicator of coagulopathy; when the value is above threshold, FFP is administered. K time and alpha angle follow and are most dependent on the availability of fibrinogen to be cleaved into fibrin while in the presence of thrombin. If indicated by K and a angle, cryoprecipitate is administered, providing a concentrated form of fibrinogen (150 to 250 mg/10 mL). MA is then noted, considering the relationship between fibrin generated during the initial phases of hemostasis and platelets via GP IIb-IIIa receptor interaction. Platelets are administered based on an MA < 54 mm, which reflects the platelets' functional contribution to clot formation. Antifibrinolytic agents have proven effective in hemorrhage during cardiac surgery and hepatic transplantation. However, both cost and morbidity associated with indiscriminant use mandate an accurate diagnosis of fibrinolysis. Of note, TEG is the only current test able to establish a diagnosis of fibrinolysis rapidly and reliably in the acutely bleeding patient.

After the tracing has reached MA, an EPL index is obtained based on the decreasing rate of clot strength. Epsilon-aminocaproic acid is indicated in the presence of significant fibrinolysis.

In summary (see Additional file 1), implementation of a goal-directed approach to post-injury coagulopathy offers the following potential benefits: (1) reduction of transfusion volumes via specific goal-directed treatment of identifiable coagulation abnormalities, (2) earlier correction of coagulation abnormalities with more efficient restoration of physiological hemostasis, (3) improved survival in the acute hemorrhagic phase due to improved hemostasis (4) improved outcomes in the later phase due to attenuation of immuno-inflammatory complications, including ARDS and MOF, and (5) improved understanding of the varied aspects of the late postinjury hypercoagulable state, potentially leading to better approaches to chemoprophylaxis and reduced thrombotic complications. Such an approach will likely help improve our understanding of the physiological basis of coagulation disturbances in the injured patient, with optimal transfusion strategies tailored to the individual patient.

## Supplementary Material

Additional file 1 **Implications of a goal directed approach to post-injury coagulopathy**. Click here for file
