# Supplementary material for: Unanswered questions in the use of blood component therapy in trauma
Source: Scand J Trauma Resusc Emerg Med. 2011 Jan 17;19:5. doi: 10.1186/1757-7241-19-5 (PMC3027129; doi:10.1186/1757-7241-19-5)
Supplement: Additional file 1 — Implications of a goal directed approach to post-injury coagulopathy. [file 1757-7241-19-5-S1.DOC]

***Implications of a goal directed approach to post-injury coagulopathy***

- ***Specific*  treatment of coagulation defects reduced transfusion volumes**
- ***Earlier* correction of coagulation defects efficient hemostasis**
- **Efficient hemostasis improved early phase survival**
- **Attenuation of immune-inflammatory mechanisms improved late phase survival**
- **Improved understanding of late hyper-coagulable state improved chemoprophylaxis and reduction of thrombotic complications**
